# Antimicrobial resistance in uropathogens in Nigeria: pathogen profiles, resistance burden, surveillance and stewardship considerations

**DOI:** 10.3389/fmicb.2026.1934847

**Published:** 2026-09-24

**Authors:** Nooruldeen Alshamaa, Jafar Ammoura, Amjad Elamin, Ramadhani Chambuso

**Affiliations:** 1 Department of Pathological Sciences, Division of Microbiology, College of Medicine, Ajman University, Ajman, United Arab Emirates; 2 Department of Global Health and Population, Harvard T. Chan School of Public Health, Harvard University, Boston, MA, United States

**Keywords:** antimicrobial resistance, secondary analysis, surveillance limitations, urinary tract infection, urine culture, uropathogens

## Abstract

**Purpose:**

We analyzed aggregated national surveillance data to describe uropathogen resistance patterns and assess limitations in the available laboratory and surveillance information from Nigeria.

**Methods:**

In a secondary data study, we retrospectively analyzed a total of 84,548 valid culture results from 26,630 patients across 25 public laboratories who participated in the national AMR report. The World Health Organization (WHO) bacterial priority uropathogens and their AMR profiles for the key urinary tract infections (UTIs) antibiotic classes were assessed.

**Results:**

More than 6,000 urine specimens were submitted for culture. The source report included both genus-level and species-level bacterial identifications. *Escherichia coli* was the most frequently reported species in each year, while *Klebsiella, Proteus, Pseudomonas, Enterococcus*, and *Staphylococcus* taxa were also recorded. Among Gram-negative bacteria, *Klebsiella* species and *Escherichia coli* showed alarming resistance to cephalosporins (80% each). *Staphylococcus aureus* showed the highest reported resistance burden, with methicillin and folate pathway inhibitor resistance reaching 82% and 89%, respectively. The descriptive assessment identified incomplete antimicrobial susceptibility testing for some pathogen-antimicrobial combinations and limitations in species-level and molecular resistance reporting. We created conceptual figures that summarize potential surveillance and stewardship considerations for clinical effectiveness.

**Conclusion:**

High reported resistance to several antimicrobial classes was observed among bacterial taxa included in the surveillance report. Our findings provide a historical descriptive summary and suggest priorities for future surveillance; to address existing national antimicrobial resistance patterns and demonstrating the effectiveness of specific clinical or policy interventions.

## 1 Introduction

Urinary tract infections (UTIs) caused by uropathogens are among the most common bacterial infections globally, with over 400 million cases annually and significant recurrence rates (Barron, 2024). In West Africa, the burden of UTIs is underestimated due to fragmented diagnostics and underreporting, with UTIs potentially accounting for over 30% of hospital-acquired infections (Melariri et al., 2024). In Nigeria, the most populous country in Africa, local surveillance reports estimate tens of thousands of UTIs each year. UTIs contribute to antimicrobial overuse, hospital readmissions, and an increased burden of antimicrobial resistance (AMR). AMR has driven repeated empirical treatment failures, especially where diagnostics are limited and substantial resistance is present (NCDC, 2023). Previous studies have explored pathogen profiles and AMR patterns; however, few have integrated uropathogen-specific resistance mapping with explicit assessment of laboratory and reporting limitations. National multi-center surveillance data may support descriptive assessment of resistance patterns and surveillance gaps (NCDC, 2023; Ajani et al., 2020).

AMR in uropathogens is fundamentally a microbiological phenomenon driven by selective pressure, genetic exchange, diagnostic limitations, and therapeutic failure. The WHO bacterial priority pathogens for UTIs such as *Escherichia coli*, *Klebsiella pneumoniae*, *Pseudomonas aeruginosa*, and *Enterococcus faecium* are frequently the causative agents of UTIs, especially in healthcare-associated infections (Bayaba et al., 2025). These organisms exhibit complex resistance mechanisms, including extended-spectrum *β*-lactamase (ESBL) production, carbapenemase activity, and biofilm formation. Uropathogenic *Escherichia coli* uses adhesins, including type 1 and P fimbriae, to colonize the urinary tract. Some strains also express toxins and other virulence factors that contribute to tissue injury, persistence, and recurrent infection (Shrestha et al., 2019; Nasrollahian et al., 2024). The capsule of *Klebsiella pneumoniae* primarily contributes to immune evasion and persistence. Antimicrobial resistance is mediated mainly through mechanisms such as *β*-lactamase and carbapenemase production, altered membrane permeability, target modification, and efflux activity (Liu et al., 2025; Xu et al., 2024). The increasing resistance of uropathogens to first-line antimicrobial treatment compromises UTI therapy and leads to prolonged infection control challenges. This promotes the spread of resistant strains and complicates the management of UTIs, which is now a global health problem (Mancuso et al., 2023; Abdalla et al., 2025; Zhou et al., 2023).

We hypothesized that the available national AMR surveillance data from Nigeria would show substantial AMR among priority uropathogens and reveal laboratory and reporting limitations that may improve the clinical utility of the available resistance data (Carlotta et al., 2024; Nedbal et al., 2024). Because the source dataset did not include objective prescribing records, dispensing data, treatment guidelines, or assessments of guideline adherence, clinical policy implementation and antimicrobial-use practices were not directly evaluated in this study.

In this secondary analysis study, we examined the existing national AMR surveillance report from Nigeria to describe priority uropathogens taxa AMR reported from urine specimens, summarize available AMR patterns, and identify limitations in laboratory identification, susceptibility testing, and surveillance reporting.

## 2 Materials and methods

### 2.1 Ethics statement

This study utilized publicly available, de-identified AMR surveillance data originally collected by the Ministry of Health of Nigeria under the Fleming Fund Regional Grant (Phase 1): https://aslm.org/wp-content/uploads/2023/07/AMR_REPORT_NIGERIA.pdf?x89467 (accessed on 17 July 2025). No individual-level data were accessed. Secondary data use complied with the Declaration of Helsinki and did not require additional ethical clearance.

### 2.2 The study design

A retrospective study was performed on the national AMR surveillance report from Nigeria for the years 2016 to 2018, similar to a published study (Osena et al., 2025). This published report was generated by the Mapping Antimicrobial Resistance and Antimicrobial Use Partnership (MAAP) consortium. It involved 25 sentinel laboratories across Nigeria with bacteriology testing capacity. This study adheres to the STROBE (Strengthening the Reporting of Observational Studies in Epidemiology) guidelines (von Elm et al., 2007).

### 2.3 Data sources and surveillance framework

The original data were collected by health authorities in Nigeria in collaboration with MAAP partners (MAAP, 2024). Surveillance activities were carried out across a national network of medical laboratories selected for their bacteriology testing capacity. A total of 25 laboratories contributed antimicrobial susceptibility testing (AST) data using WHONET software, a standardized microbiology data management tool (Dikkatwar and Vaghasiya, 2023). Data collection involved trained field teams retrieving laboratory records from both paper-based and digital systems. Where feasible, these records were linked with hospital databases to include patient demographics and clinical metadata (e.g., age, sex, and specimen type). The surveillance focused on human health, and isolates were obtained from urine and other clinical specimens. In the participating laboratories, 11 used automated systems for bacterial identification, while 14 laboratories used manual methods. This variability in identification approach may introduce some variability in species-level accuracy. However, all sites reported compliance with CLSI AST standards, and these data provide the best available multi-laboratory AMR estimates for the study period.

Nevertheless, the source report did not provide sufficient year-specific information to confirm that laboratory participation, specimen composition, bacterial identification methods, AST panels, tested-isolate denominators, and missing-data structures remained consistent between 2016, 2017, and 2018. Cross-year comparisons were therefore treated as descriptive and were not interpreted as confirmed temporal resistance trends.

The uropathogens reported from culture results include the following:

#### 2.3.1 Positive cultures with no species identification

(i) *Klebsiella* species
(ii) *Proteus* species
(iii) *Pseudomonas* species
(iv) *Enterococcus* species
(v) *Staphylococcus* species

#### 2.3.2 Positive cultures with species identification

(i) *Klebsiella aerogenes*
(ii) *Klebsiella oxytoca*
(iii) *Klebsiella pneumoniae*
(iv) *Escherichia coli*
(v) *Escherichia fergusonii*
(vi) *Proteus hauseri*
(vii) *Proteus mirabilis*
(viii) *Proteus vulgaris*
(ix) *Pseudomonas aeruginosa*
(x) *Pseudomonas fluorescens*
(xi) *Pseudomonas stutzeri*
(xii) *Enterococcus faecalis*
(xiii) *Staphylococcus aureus*

### 2.4 Inclusion criteria and data management

Only isolates from the above bacterial groups with valid AST results were included in the analysis. In accordance with the WHO Global Antimicrobial Resistance Surveillance System (GLASS) guidelines and Clinical and Laboratory Standards Institute (CLSI) M39-A4 recommendations, only the first isolate per patient per year was retained to avoid duplicate counting (Pierce and Mathers, 2022; WHO, 2022). In cases where unique patient identifiers were not available, all isolates were retained and interpreted with appropriate caution. AST interpretations followed CLSI criteria. Where zone diameters or minimum inhibitory concentrations were available, WHONET was used to manage and standardize susceptibility data for surveillance analysis (Dikkatwar and Vaghasiya, 2023; Banibella et al., 2025; Kowalska-Krochmal and Dudek-Wicher, 2021).

### 2.5 Data extraction from the report

In this study, we manually extracted relevant pathogen-specific data from the national report, including the following:

- (i) Total number of participating laboratories.
- (ii) Collected specimens per year.
- (iii) Source of collected specimens.
- (iv) Specimen types.
- (v) Valid cultures.
- (vi) Positive and negative cultures.
- (vii) Species-level breakdown (e.g., *E. coli*, *K. pneumoniae*, *P. aeruginosa*, etc.).
- (vii) AST results for antibiotics tested.
- (ix) Patient demographics (age and sex).

These variables were compiled into a structured dataset with multiple spreadsheets for different variables (see Supplementary material) to enable consistent data analysis. Species were identified where possible, while others were recorded generically as *Klebsiella* spp., *Escherichia* spp., *Proteus* spp., *Pseudomonas* spp., or *Enterococcus* spp., according to how they appeared in the original report. AMR data were available for multiple antibiotics from anti-biograms and figures for descriptive analysis of year-specific resistance patterns and common resistant pathogen-antimicrobial combinations.

### 2.6 AST and quality control

AST was conducted using both disk diffusion and MIC panels, depending on each laboratory’s capacity. The primary interpretive standard applied was CLSI criteria. Quality control strains were widely used for routine AST quality control. External Quality Assessment (EQA) was implemented across participating laboratories through a structured proficiency testing program coordinated by MAAP. The Fleming Fund supported laboratories were encouraged to participate in periodic EQA rounds, and corrective actions were documented for all laboratories with substandard performance (Osena et al., 2025; Kostyanev et al., 2025). However, details on the proportion of laboratories consistently participating in EQA or their inter-laboratory performance were not systematically reported.

### 2.7 AMR calculation

From the original report, AMR rates were derived from positive cultures with available AST results. AMR rates were calculated as the proportion of non-susceptible isolates (intermediate or resistant) relative to the total number of tested isolates within a single calendar year.


$$\begin{array}{l} \mathrm{AMR}\;\text{Rate}(\%)=\frac{\text{Number of non}-\text{susceptible isolates}}{\begin{array}{l} \text{Number of isolates tested for}\\ \;\text{antimicrobial susceptibility} \end{array}}\times 100\;\\ \left(95\%\text{Confidence Interval}\right) \end{array}$$

Where

- Non-susceptible isolates = resistant + intermediate.
- Tested isolates = all isolates subjected to AST.

However, as our study analyzed the national AMR surveillance report, only aggregated proportions of non-susceptible isolates were provided in the anti-biogram figures and tables. The raw stratified susceptibility/intermediate/resistant (S/I/R) counts were not available for extraction. Hence, S/I/R data could not be reconstructed from the published report. Our interpretation remains aligned with the WHO GLASS reporting standards, which combine intermediate and resistant isolates for surveillance purposes.

### 2.8 Statistical analysis

Statistical analyses and figure visualizations were performed using R version 4.3.2 and Python version 3.11. Missing data accounted for less than 1% and were handled using complete-case analysis. Valid culture results were summarized using descriptive statistics across demographic groups and years. Categorical variables are presented as frequencies and percentages, and culture positivity rates are reported with 95% confidence intervals. No inferential hypothesis testing was performed.

Logistic regression, multivariable modeling, and formal temporal resistance-trend testing were not performed because the publicly available report did not provide patient-level data, complete year-specific pathogen-antimicrobial denominators, or raw susceptible, intermediate, and resistant counts. Applying these models to aggregated proportions would risk ecological inference and potentially misleading estimates.

## 3 Results

### 3.1 Demographics and culture characteristics

Among 84,548 valid cultures analyzed, the majority were obtained from female patients (55.6%). The observed culture positivity proportion was higher among females than males (35.1% vs. 28.3%, respectively; Table 1). Culture positivity increased steadily with age, reaching 40.0% in those aged >65 years. Although the largest number of cultures originated from adults aged 18–49 years, their positivity rate was moderate (31.8%) compared with older groups. Reported culture positivity proportions were 28.5% in 2016, 33.1% in 2017, and 34.6% in 2018. These year-specific differences were not interpreted as confirmed temporal trends because consistency in laboratory participation, specimen composition, and reporting denominators could not be fully assessed.

**Table 1:** Distribution of valid microbiological cultures by gender, age group, and year.

Variable	Valid cultures, *n*	Positive cultures, *n*	Positive cultures with AST, *n*	Culture positivity (%)	95% CI
Gender					
Male	37,550	10,618	10,160	28.3	27.8–28.7
Female	46,997	16,517	13,803	35.1	34.7–35.6
Age group					
<1 year	9,689	2,832	2,711	29.2	28.3–30.1
1–17 (yrs)	20,476	4,902	4,526	23.9	23.4–24.5
18–49 (yrs)	29,336	9,323	7,629	31.8	31.2–32.3
50–65 (yrs)	5,177	1,753	1,630	33.9	32.6–35.2
Above 65 (yrs)	4,743	1,898	1,806	40.0	38.6–41.4
Unknown age	15,127	6,427	5,661	42.5	41.7–43.3
Year					
2016	26,896	7,678	6,532	28.5	28.0–29.1
2017	32,134	10,640	9,501	33.1	32.6–33.6
2018	25,518	8,817	7,930	34.6	34.0–35.1

95% confidence intervals were calculated using the Wilson method for culture positivity percentages.

### 3.2 Diagnostic yield, specimen distribution, and year-specific pathogen patterns across surveillance laboratories

Across the 25 sentinel laboratories surveyed, marked heterogeneity was observed in the microbiological diagnostic yield and reporting completeness of cultures (Figure 1a). While most laboratories reported a predominance of negative cultures, a concerning proportion of positive cultures lacked antimicrobial susceptibility testing (AST), particularly at Murtala Muhammad and Minna laboratories. Only a few centers consistently linked culture positivity with AST reporting. Among the top 10 leading specimen types, urine was the most frequently processed specimen across all years, peaking in 2018 (Figure 1b). Multiple uropathogen taxa were identified between 2016 and 2018, and *Escherichia coli* was the most frequently isolated species each year (Figure 1c). The reported number of *Klebsiella* species isolates was 922 in 2016 and 1,245 in 2018. Among species-level identifications, the highest reported annual count of *Klebsiella pneumoniae* occurred in 2017, with 851 isolates. Other frequently reported pathogens included *Pseudomonas aeruginosa*, *Enterococcus* species, and *Proteus mirabilis*, while *Escherichia fergusonii*, *Pseudomonas stutzeri*, and *Proteus hauseri* were rarely detected.

**Figure 1:**
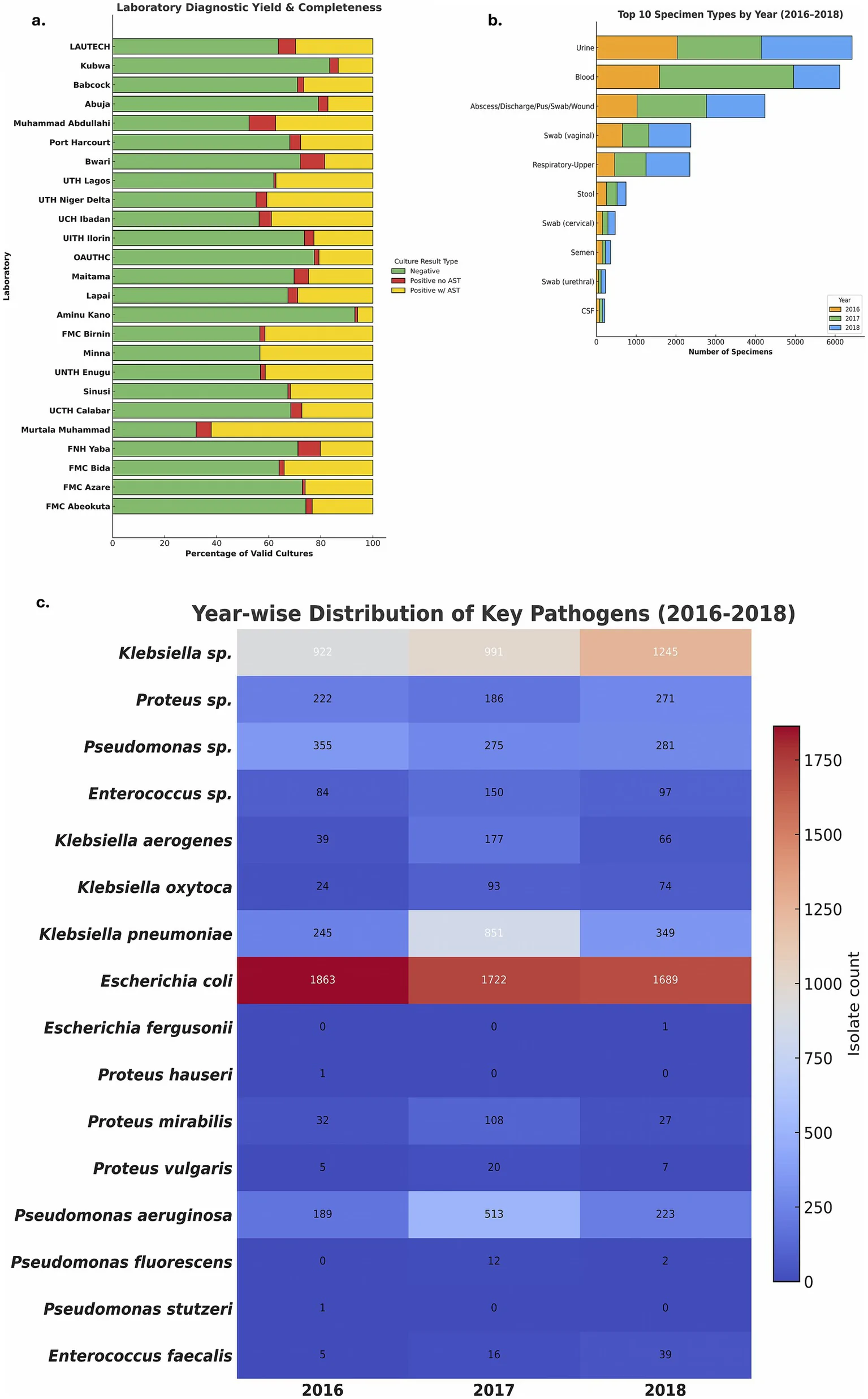
Laboratory diagnostic yield, specimen distribution, and year-specific pathogen patterns. **(a)** Laboratory-level distribution of valid urine cultures categorized by diagnostic yield, showing inter-laboratory variability and several sites with culture-positive specimens lacking AST data. **(b)** Bar chart showing the top 10 specimen types processed annually. Urine remained the most common collected specimen type across all years. **(c)** Heatmap of year-specific isolate counts for key uropathogens. *Escherichia coli* consistently ranked as the leading reported species during the surveillance period.

### 3.3 Yearly AMR profiles across drug classes and specific uropathogen species

Given the clinical reliance on empiric therapy for uropathogens in low-resource settings, we constructed a comparative AMR gradient to summarize reported resistance proportions by pathogen-drug class combination for 2016 (Figure 2). Among Gram-negative bacteria, *Klebsiella* spp. and *Escherichia coli* showed high reported resistance to cephalosporins (80% each). *Proteus* species also exhibited high resistance to folate pathway inhibitors (91%), a concern for empirical treatment in urinary infections. No vancomycin resistance was detected in the Gram-positive bacteria tested, including *Staphylococcus aureus* and *Enterococcus faecalis*. *Staphylococcus aureus* and other *Staphylococcus species* displayed resistance above 80% to methicillin, consistent with high reported methicillin resistance among *Staphylococcus* taxa.

**Figure 2:**
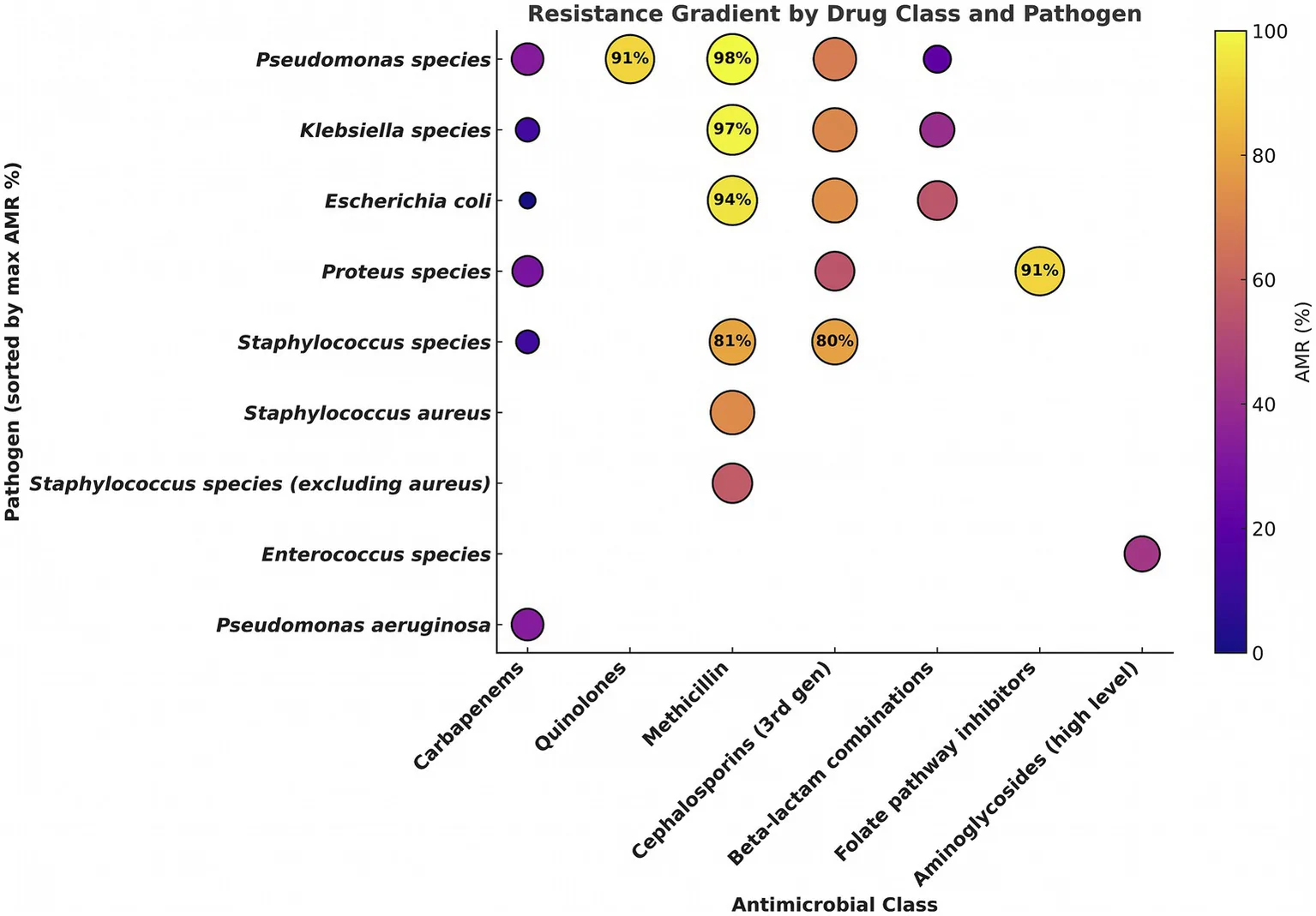
Resistance gradient of priority uropathogens across antimicrobial classes in 2016. *Proteus* species showed 91% resistance to folate pathway inhibitors. Resistance to carbapenems and aminoglycosides remained comparatively low across reported uropathogens. Orange-outlined bubbles denote AMR ≥ 80%, and gradients summarize potential therapeutic constraints for UTIs during the surveillance period. “Beta-lactam combinations” refers to agents tested with beta-lactam/beta-lactamase inhibitor pairings, principally amoxicillin-clavulanic acid and piperacillin-tazobactam.

In 2017, *Klebsiella aerogenes* exhibited the highest reported resistance levels, with 91% to second-generation cephalosporins and 83% to quinolones, reflecting substantial beta-lactam and fluoroquinolone resistance (Figure 3). *Escherichia coli* showed high reported resistance to tetracyclines, reaching 88% in the available 2017 data. Resistance among *Klebsiella* and *Staphylococcus* species was observed across several tested drug classes, consistent with broad resistance patterns reported for these taxa (Ballén et al., 2021; Gebremeskel et al., 2023; Barman et al., 2024).

**Figure 3:**
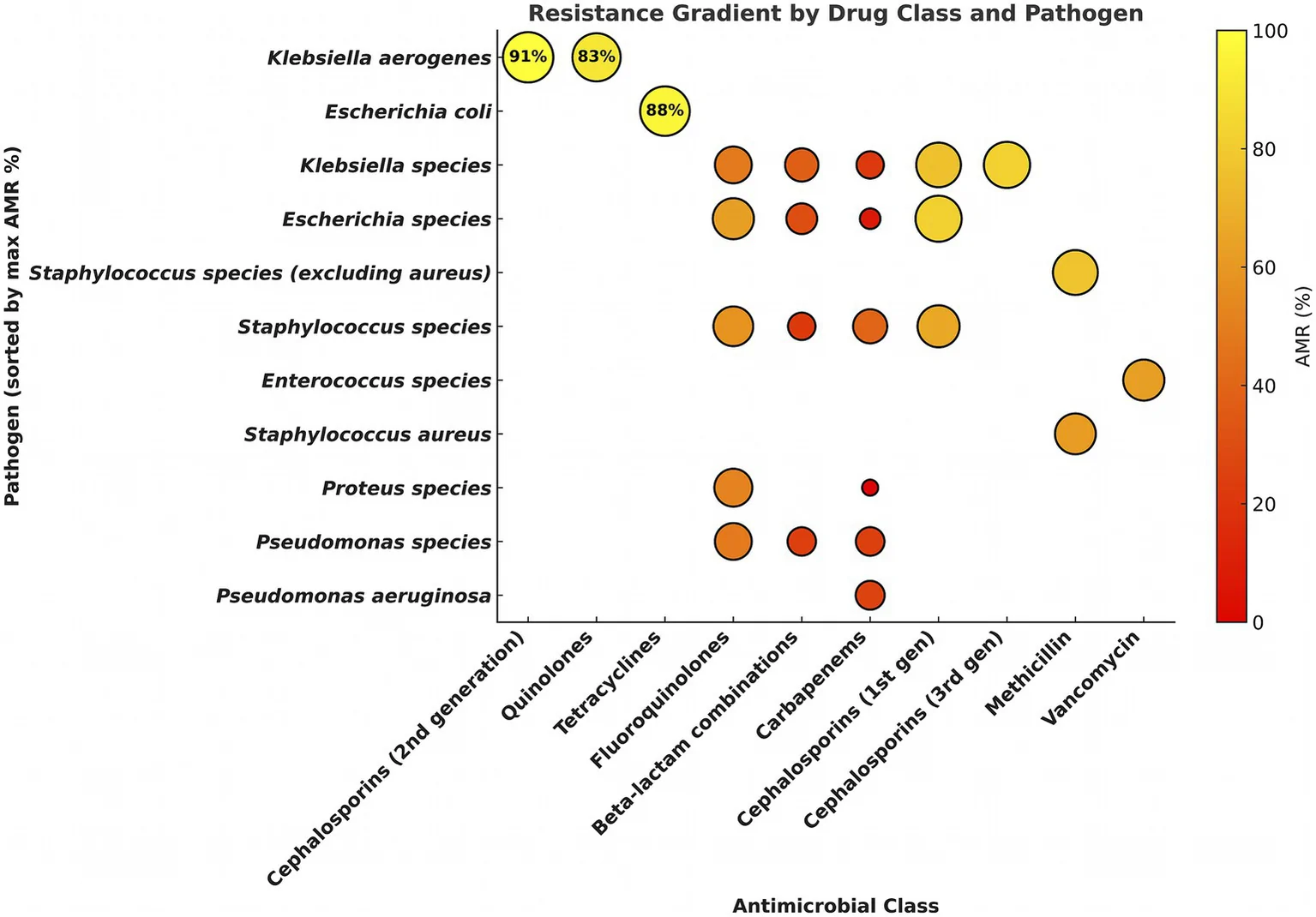
Resistance gradient of priority uropathogens to diverse antimicrobial classes in 2017. *Klebsiella aerogenes* showed high reported resistance, with 83 and 91% to quinolones and second-generation cephalosporins, respectively. *Escherichia coli* also exhibited high resistance to tetracyclines, reaching 88% in the available data. *Pseudomonas aeruginosa* displayed comparatively lower reported AMR rates. Black-bordered bubbles indicate resistance rates ≥80%. “Beta-lactam combinations” refers to agents tested with beta-lactam/beta-lactamase inhibitor pairings, principally amoxicillin-clavulanic acid and piperacillin-tazobactam.

In 2018, *Staphylococcus aureus* showed a high reported resistance burden, with methicillin and folate pathway inhibitor resistance reaching 82% and 89%, respectively (Figure 4). Pseudomonas species and *Escherichia coli* also showed high resistance profiles, particularly to tetracyclines and folate pathway inhibitors (86 and 85%, respectively), highlighting treatment challenges documented during the surveillance period. High resistance to first-generation cephalosporins among Staphylococcus species and *Escherichia coli* suggests reduced empirical coverage in the reported dataset.

**Figure 4:**
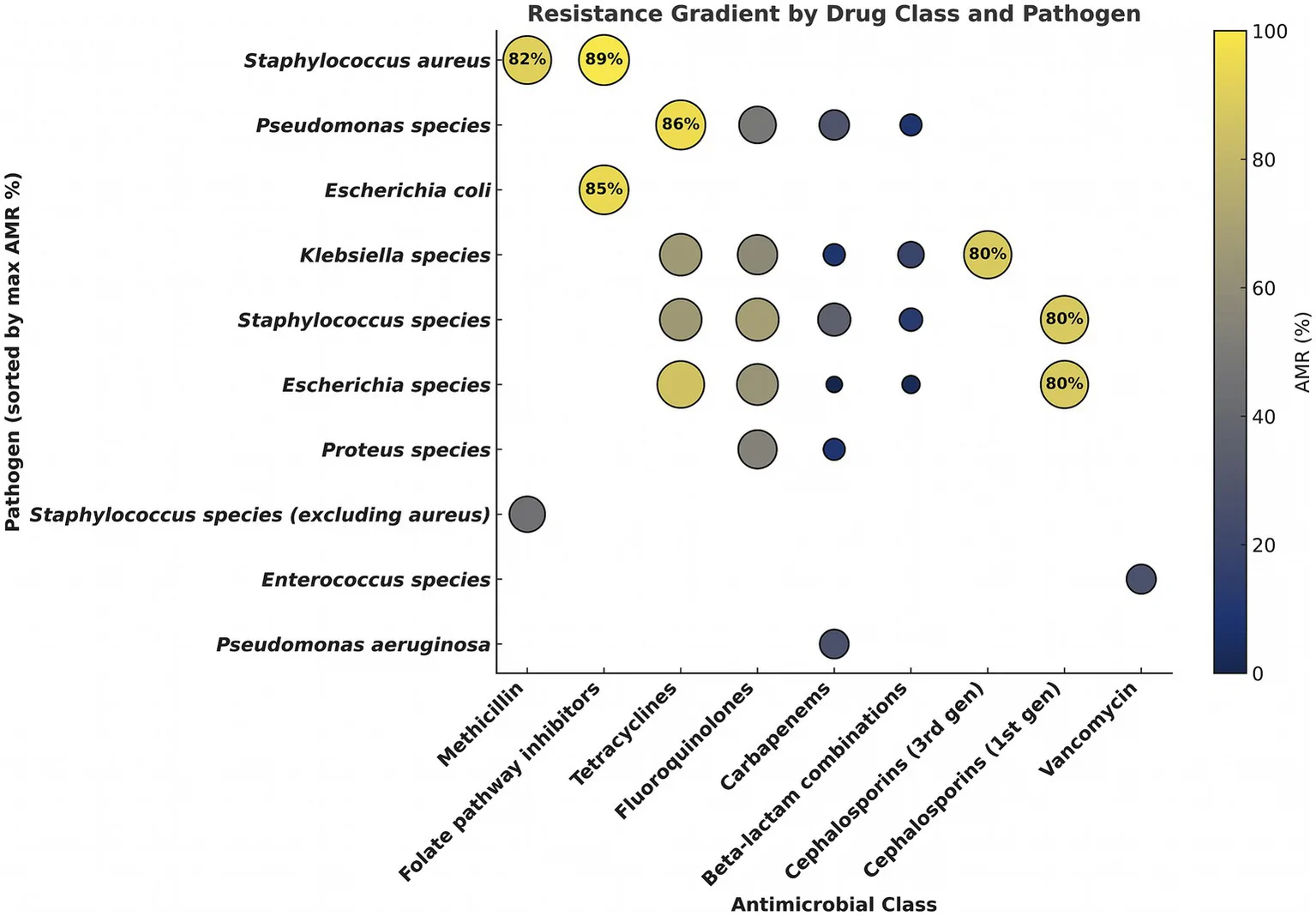
AMR gradients across uropathogens and drug classes in 2018. *Staphylococcus aureus* showed high methicillin and folate pathway inhibitor resistance, while *Escherichia coli* showed high resistance to folate pathway inhibitors. *Pseudomonas* species showed concerning resistance to tetracycline. *Klebsiella* species had 80% resistance to third-generation cephalosporins. Black-bordered bubbles indicate resistance rates ≥80%.

### 3.4 Assessment of laboratory and surveillance limitations

Analysis of the secondary surveillance data identified several laboratory and reporting limitations that may reduce the clinical utility of the available anti-biograms and local resistance mapping. These included incomplete antimicrobial susceptibility testing for some pathogen-antimicrobial combinations, inconsistent species-level identification, and limited availability of molecular resistance data. However, the source dataset did not include objective prescribing records, pharmacy dispensing data, institutional treatment guidelines, or assessments of guideline adherence. Therefore, antimicrobial-use practices, feedback to antimicrobial stewardship teams, and the presence of clinical policy gaps could not be directly evaluated (see Figure 5).

**Figure 5:**
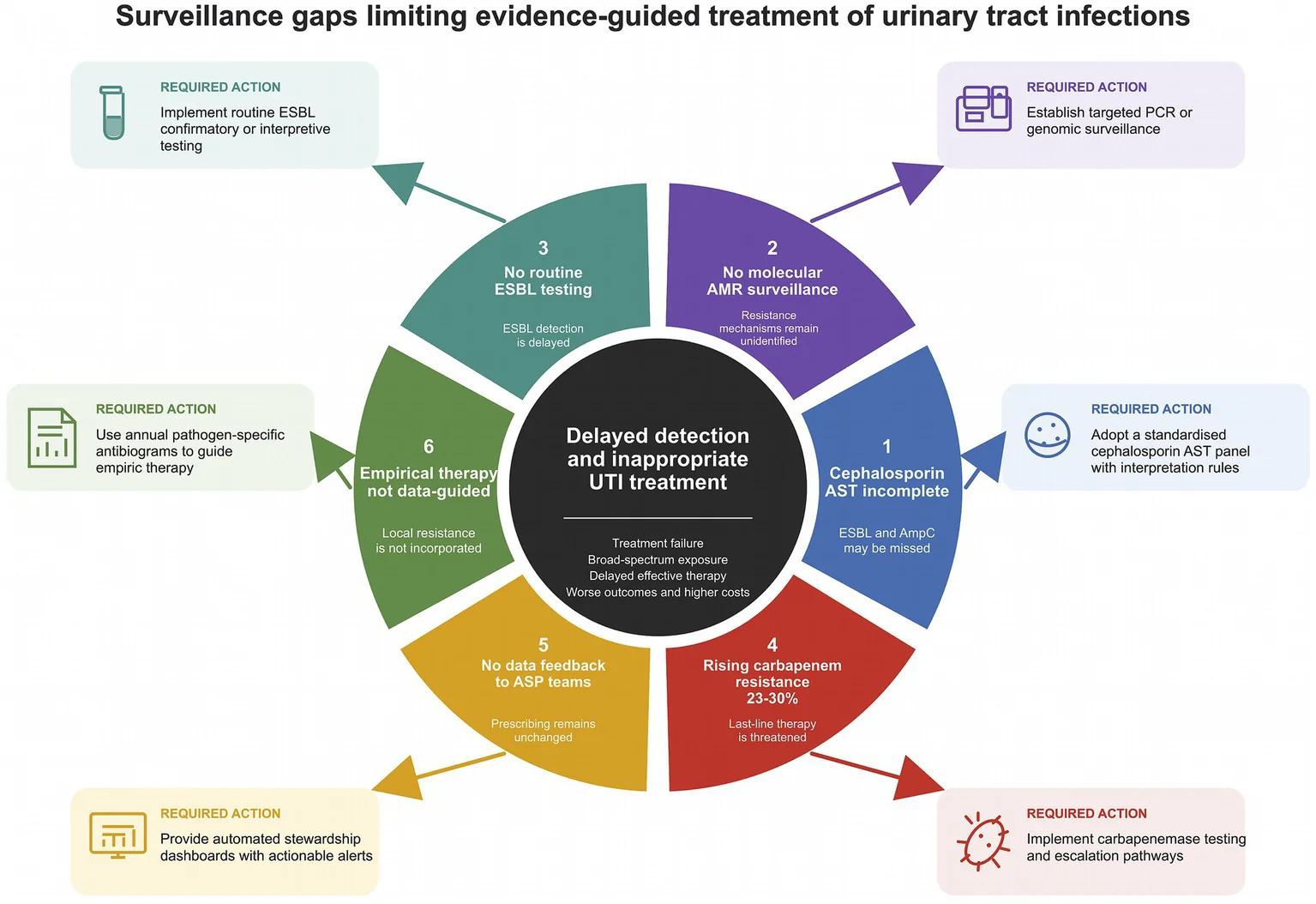
Visualizing laboratory and surveillance limitations relevant to UTI AMR interpretation. The figure summarizes reported limitations such as incomplete molecular AMR characterization, incomplete ESBL-related information, incomplete AST reporting for some pathogen-antimicrobial combinations, and limited linkage between microbiology data and stewardship evaluation. These limitations may reduce the clinical interpretability of the available uropathogen resistance data and identify priorities for future surveillance strengthening. AST, antimicrobial susceptibility testing; ASP, antimicrobial stewardship program; ESBL, extended-spectrum beta-lactamase.

### 3.5 Proposed pathogen-specific surveillance and stewardship considerations

Based on the resistance patterns documented during the 2016–2018 surveillance period, we developed a hypothesis-generating map of potential pathogen-specific laboratory and stewardship considerations. The map links selected uropathogens with possible responses, including enhanced resistance alerts, improved species-level identification, expanded molecular testing, and review of empirical treatment guidance. These links represent proposed recommendations and should not be interpreted as evidence that the suggested interventions were absent from existing national or institutional policies. Confirmation would require objective assessment of prescribing records, dispensing data, treatment guidelines, and guideline adherence (see Figure 6).

**Figure 6:**
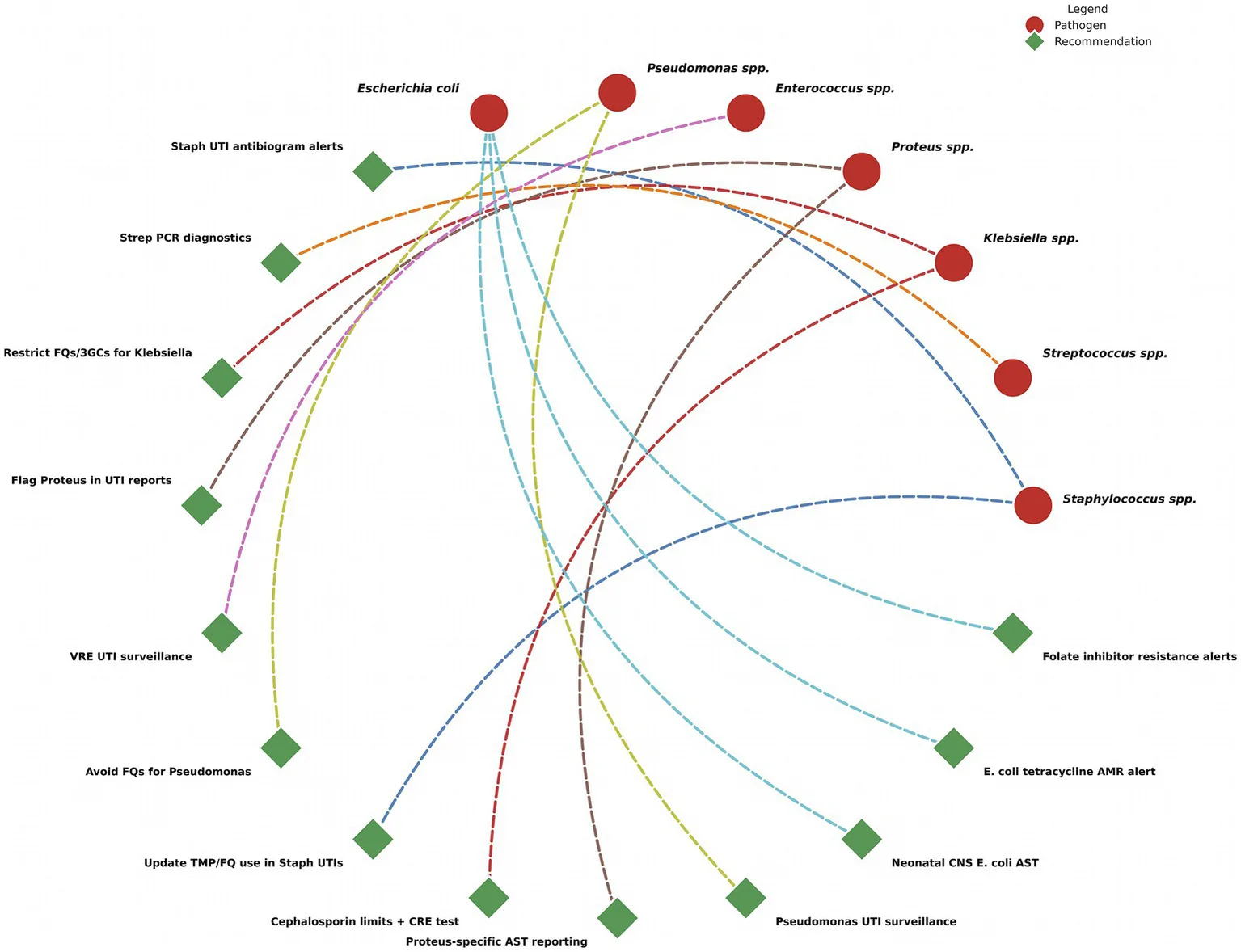
Proposed pathogen-specific surveillance and stewardship considerations. Arced dashed lines connect selected pathogens to potential responses, with arc color mapped to specific taxa. For example, *Escherichia coli* was linked to enhanced monitoring of tetracycline and folate pathway inhibitor resistance, while *Pseudomonas* species were linked to strengthened surveillance and careful review of fluoroquinolone susceptibility before empiric-use decisions. The map is hypothesis-generating and requires validation with recent resistance data, prescribing records, guideline review, and patient-level outcomes before policy implementation.

## 4 Discussion

This study presents a multi-center secondary analysis of more than 84,000 valid clinical cultures collected between 2016 and 2018 across 25 laboratories in Nigeria. Focusing on bacterial taxa reported from urine specimens, we summarized year-specific AMR patterns, pathogen-specific resistance burdens, and laboratory and surveillance limitations affecting the interpretation of the available data. We also integrated resistance-gradient visualizations with proposed conceptual maps of potential laboratory and stewardship considerations. These findings highlight the importance of strengthening the use of microbiological surveillance data to support future evaluation of UTI management and antimicrobial stewardship practices.

This study identified important limitations in the laboratory characterization and surveillance of bacterial taxa reported from urine specimens in Nigeria. High resistance to several antimicrobial classes, particularly folate-pathway inhibitors and cephalosporins, was documented in the available 2016–2018 data (Mareș et al., 2023; Benea et al., 2025). Incomplete AST reporting, limited molecular resistance information, and inconsistent species-level identification reduced the interpretability of the surveillance findings. However, because the dataset did not include objective prescribing records, treatment guidelines, or assessments of guideline adherence, the study could not directly evaluate prescribing practices or confirm clinical policy gaps. The findings instead suggest areas that may warrant further investigation using more recent and clinically detailed data (Rodriguez et al., 2025; Mao et al., 2023).

The findings may help identify priorities for future surveillance and laboratory evaluation in Nigeria, including more complete antimicrobial susceptibility testing, improved species-level identification, and targeted molecular resistance characterization (Miller et al., 2018; Miller et al., 2024). However, because the analysis was based on aggregated historical surveillance data, the proposed laboratory and stewardship considerations should be regarded as hypothesis-generating. More recent resistance data, prescribing and dispensing records, guideline-adherence assessments, and patient-level outcomes would be required before these findings could inform changes to current treatment guidelines or national AMR policy.

*Escherichia fergusonii* was an uncommon organism reported in the source dataset. It was retained for completeness because it appeared in the MAAP national AMR report; however, its clinical significance as a urinary pathogen could not be established from the aggregated data. Interpretation therefore focused primarily on established uropathogens, particularly *Escherichia coli*, while *E. fergusonii* was treated as a minor descriptive finding.

The methicillin resistance reported among *Staphylococcus taxa* exceeded 80% and was higher than estimates reported in previous UTI-AMR studies in Nigeria (Ezeh et al., 2023; Ike et al., 2016). Differences may reflect variation in study populations, specimen selection, laboratory methods, tested-isolate denominators, or reporting practices. Unlike the previous studies, the present analysis also summarized laboratory and surveillance limitations and presented a proposed conceptual map of possible stewardship considerations. This mapping should not be interpreted as a validated gap-analysis framework or as evidence of deficiencies in existing antimicrobial-use policies.

From a global health microbiology perspective, the findings contribute a historical, multi-center description of AMR among uropathogen taxa reported in Nigeria, a setting that remains underrepresented in many global AMR summaries (Ogunleye et al., 2022). The high resistance proportions observed for several frontline antimicrobial classes are consistent with concerns reported by global surveillance initiatives such as GLASS (Ajulo and Awosile, 2024; Abdullah et al., 2023). However, the present analysis should be interpreted as a secondary descriptive assessment rather than a direct evaluation of stewardship practice or policy performance. The structured pathogen-resistance mapping may be useful as a framework for generating surveillance priorities and designing future studies that integrate microbiology data, prescribing information, guideline review, and patient outcomes in low-resource settings (Gulumbe et al., 2022; Cella et al., 2023; Luong Nguyen et al., 2025).

### 4.1 The strengths of this study

(i) A large number of cultures from 25 laboratories was included, providing broad descriptive coverage of participating surveillance sites during 2016–2018.
(ii) A structured descriptive assessment was used to summarize pathogen-drug class resistance patterns and limitations in the available surveillance information.
(iii) The inclusion of data from three consecutive years (2016–2018) enabled description of year-specific AMR patterns among reported uropathogen taxa.
(iv) The focus was on WHO bacterial priority uropathogens, enhancing relevance to global microbiology and AMR surveillance goals.
(v) The assessment highlighted laboratory and reporting limitations, including incomplete ESBL-related information and incomplete species-level identification for some uropathogens.
(vi) Proposed visual summaries were used to organize laboratory limitations and potential surveillance considerations in an accessible format.
(vii) The use of a publicly available national surveillance report supports transparent secondary analysis and highlights the value and limitations of existing AMR reporting systems.

#### 4.1.1 Limitations of the secondary dataset

(i) The data were collected between 2016 and 2018 and may not reflect current pathogen distributions or antimicrobial resistance patterns.
(ii) Urine-specific culture results could not always be separated from the total clinical culture dataset.
(iii) AST results were incompletely reported for some pathogen-antimicrobial combinations, and complete susceptible, intermediate, and resistant counts were unavailable.
(iv) Several organisms were reported only at genus level, limiting species-specific interpretation.
(v) The source dataset lacked objective prescribing, dispensing, and guideline-adherence data. Therefore, antimicrobial misuse or clinical policy gaps could not be confirmed.
(vi) The source report did not provide sufficient information to confirm consistent laboratory participation, specimen composition, identification methods, AST panels, tested-isolate denominators, or missing-data structures across 2016–2018. Apparent year-to-year differences may therefore reflect changes in reporting or testing practices rather than true epidemiological trends.

#### 4.1.2 Limitations of the present study

- (i) This study was a retrospective descriptive analysis of a publicly available surveillance report and was limited to the variables and classifications reported in the original dataset.
- (ii) Patient-level clinical outcomes, including treatment failure, complications, and mortality, could not be assessed.
- (iii) We could not verify the original laboratory records or perform additional molecular testing, such as ESBL or carbapenemase gene confirmation.
- (iv) The descriptive design does not allow causal conclusions or confirmation that changes across years represented true epidemiological trends rather than differences in sampling or laboratory reporting.

Despite these potential limitations, our study provides the largest multi-center, multi-year descriptive summary of uropathogen resistance patterns reported from one of the low income countries. The scale, temporal scope, and organism-specific analysis help address knowledge gaps in regional AMR epidemiology, particularly for WHO priority bacterial taxa such as *Klebsiella*, *Escherichia coli*, and *Pseudomonas*. The findings support the need for strengthened laboratory reporting, more complete AST data, and future studies that can link microbiological surveillance with prescribing, guideline adherence, and patient outcomes.

## 5 Conclusion

High reported AMR across key drug classes for uropathogens such as *Klebsiella*, *Escherichia coli*, and *Staphylococcus species* species suggests important therapeutic constraints in the 2016–2018 surveillance dataset from Nigeria. These findings provide a historical microbiological summary that can support diagnostic stewardship planning, identify surveillance priorities, and guide the design of future studies using more recent resistance data, prescribing records, guideline review, and patient-level outcomes. Strengthening AMR surveillance, molecular diagnostic capacity, AST completeness, and feedback systems for uropathogens remains important for Nigeria’s microbiology infrastructure and for future UTI stewardship evaluation.

## Data Availability

The datasets used for the analyses in this study are available in the Supplementary material.
